# Design of a 900 MHz Dual-Mode SWIPT for Low-Power IoT Devices

**DOI:** 10.3390/s19214676

**Published:** 2019-10-28

**Authors:** Hamed Abbasizadeh, Sang Yun Kim, Behnam Samadpoor Rikan, Arash Hejazi, Danial Khan, Young Gun Pu, Keum Cheol Hwang, Youngoo Yang, Dong In Kim, Kang-Yoon Lee

**Affiliations:** 1 Department of Electrical and Computer Engineering, University of California at San Diego, La Jolla, CA 92093, USA; habbasizadeh@ucsd.edu; 2 Department of Electrical and Computer Engineering, Sungkyunkwan University, Suwon 16419, Korea; ksy0501@skku.edu (S.Y.K.); arash@skku.edu (A.H.); danialkhan@skku.edu (D.K.); hara1015@skku.edu (Y.G.P.); khwang@skku.edu (K.C.H.); yang09@skku.edu (Y.Y.); dikim@skku.ac.kr (D.I.K.); 3 Nanoelectronics Group, Department of Informatics, University of Oslo, 0316 Oslo, Norway; behnam@ifi.uio.no

**Keywords:** IoT devices, information decoding, PAPR, RF energy harvester, reconfigurable, simultaneous wireless information and power transceiver

## Abstract

This paper presents a duty cycle-based, dual-mode simultaneous wireless information and power transceiver (SWIPT) for Internet of Things (IoT) devices in which a sensor node monitors the received power and adaptively controls the single-tone or multitone communication mode. An adaptive power-splitting (PS) ratio control scheme distributes the received radio frequency (RF) energy between the energy harvesting (EH) path and the information decoding (ID) path. The proposed SWIPT enables the self-powering of an ID transceiver above 20 dBm input power, leading to a battery-free network. The optimized PS ratio of 0.44 enables it to provide sufficient harvested energy for self-powering and energy-neutral operation of the ID transceiver. The ID transceiver can demodulate the amplitude-shift keying (ASK) and the binary phase-shift keying (BPSK) signals. Moreover, for low-input power level, a peak-to-average power ratio (PAPR) scheme based on multitone is also proposed for demodulation of the information-carrying RF signals. Due to the limited power, information is transmitted in uplink by backscatter modulation instead of RF signaling. To validate our proposed SWIPT architecture, a SWIPT printed circuit board (PCB) was designed with a multitone SWIPT board at 900 MHz. The demodulation of multitone by PAPR was verified separately on the PCB. Results showed the measured sensitivity of the SWIPT to be −7 dBm, and the measured peak power efficiency of the RF energy harvester was 69% at 20 dBm input power level. The power consumption of the injection-locked oscillator (ILO)-based phase detection path was 13.6 mW, and it could be supplied from the EH path when the input power level was high. The ID path could demodulate 4-ASK- and BPSK-modulated signals at the same time, thus receiving 3 bits from the demodulation process. Maximum data rate of 4 Mbps was achieved in the measurement.

## 1. Introduction

Energy-limited wireless devices in Internet of Things (IoT) and wireless sensor networks (WSN) are typically powered by batteries with limited lifetime. Although their replacement and recharging can extend the operation of the system for a certain amount of time, it usually results in high costs. In recent times, radio frequency (RF) energy harvesting (EH) has potentially overcome the need for battery replacement and recharging, and it is now being used as the power supply of low-power wireless devices [1,2,3]. RF signals carrying both information and power can be utilized for information and power transfer at the same time. Simultaneous wireless information and power transfer (SWIPT) is attracting growing attention. It is becoming a promising source of power supply and for delivery of information on wireless devices in the IoT and WSN.

The concept of SWIPT was initially introduced in [4], in which the trade-off between power and information transfer was discussed. Later, in [4,5,6], the authors assumed the receivers to simultaneously harvest energy and decode information from the same signal. In [7], the authors came to the conclusion that the merging of energy harvesting hardware and decoding blocks is difficult. Hence, recent literature has introduced two well-known practical approaches for receiver architecture: time-switching (TS) receiver [7,8,9,10,11,12] and power-splitting (PS) receiver [7,11,13,14,15,16]. In both receivers, the received RF signal needs to be split into two parts: one for energy harvesting and the other for information decoding (ID). In the TS technique, the received RF signal is used either for EH or ID in one time slot. On the other hand, the PS scheme splits the received RF signal into two paths—EH path and ID path—using the power-splitter component. The PS scheme is more complex than the TS scheme; however, it allows instantaneous SWIPT, namely, the received RF signal is used for both EH and ID in one time slot. In this paper, we propose a dual-mode (single-tone and multitone waveforms) SWIPT system based on the adaptive power-splitter (APS) scheme. Moreover, we adopt duty cycle operation for the dual-mode SWIPT with self-powering, which considers a nonlinear EH model for both single-tone and multitone waveforms. The purpose of the dual-mode operation is that multitone waveforms provide higher power conversion efficiency (PCE) with nonlinear energy harvesting than single-tone waveform at low-input power level. However, single-tone-based amplitude and phase modulation may not be used with multitone waveforms. To tackle this difficulty, we propose a peak-to-average power ratio (PAPR)-based SWIPT [17,18], a technique that uses multitone waveforms and their distinct levels of PAPR to convey information. 

The PAPR-based SWIPT achieves higher PCE, which increases the operational range while requiring less energy and low complexity for ID. It is well known in the literature on SWIPT that TS-based SWIPT is simple to realize, while PS-based SWIPT performs better in terms of rate–energy trade-off than the former. This is because the former is a specific realization of the latter. The key element in a SWIPT system for RF energy harvesting is the energy harvesting circuit. It primarily consists of an RF–direct current (DC) converter that converts the received RF power into DC power. Efficiency in the RF–DC converter is very critical in defining the overall SWIPT efficiency [10,11,12,13,18,19,20,21]. Harvested RF energy is generally very small and may not be sufficient enough to operate active RF communication. Therefore, harvesting energy will render prolongation of the battery lifetime in active RF wireless communication systems. For batteryless communication devices, we should be able to design an ultra-low-power communication system that can efficiently operate under the limited harvested power. In SWIPT, the received RF power is used for dual purpose: EH and ID. The existing RF communication design consumes much more power compared to harvested RF energy. It is well known in the literature that most of the power in existing RF communications is consumed by oscillators, which generate sinusoidal carrier signals for modulation of data. This high-power consumption hinders application of energy harvesting wireless communications. Therefore, a low-power transceiver architecture without the oscillator is considered to be the prerequisite for realizing the self-powered SWIPT considered herein. For this, we propose a high-efficiency energy harvesting circuit along with ultra-low-power communication transceiver design in order to achieve the required trade-off between the harvested energy and the achievable data rate [22,23,24,25,26,27,28,29,30,31,32,33,34,35,36,37,38,39].

The rest of the paper is organized as follows. Section 2 explains the architecture and building blocks of the proposed RF energy harvester. Simulations and experimental results are discussed in Section 3. Section 4 provides concluding remarks.

## 2. System Architecture and Building Blocks

### 2.1. System Model

**Figure 1:**
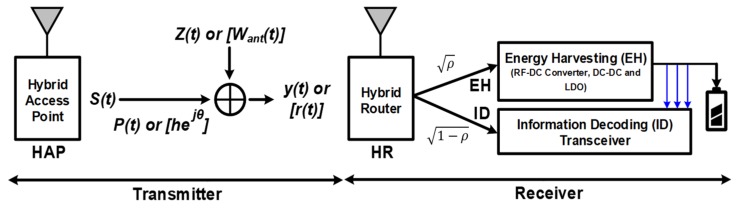
The concept of simultaneous wireless information and power transceiver (SWIPT).

Figure 1 shows the concept of SWIPT. The hybrid access point (HAP) transmits the energy signal *s*(*t*), which carries modulated data information. The channel fading is represented by $he^{j\theta }$. At the receiver side, *y*(*t*) is the received signal containing the channel noise *z*(*t*) (*W_ant_*(*t*)). The received signal is used for both EH and ID processing [4,5,6,7,8,9,10,11]. For the SWIPT system, a transmitter generates single-tone and multitone waveforms. The transmitter selects a waveform depending on the received power and feedback information from the receiver. The transmitter and receiver are jointly designed based on single-tone transmission with M-ary amplitude-shift keying (ASK) and multitone transmission with PAPR modulation. At the receiver, the received signal is decoded jointly using both coarse and fine amplitude paths. The former maps to coarse energy levels (e.g., high/low amplitude levels), while the latter maps to fine constellation points associated with the energy level. By virtue of the nonlinear rectification process, the PCE can be enhanced using multitone waveforms. Furthermore, the PAPR-based information transmission facilitates low-power ID using simple PAPR measurements [18].

**Figure 2:**
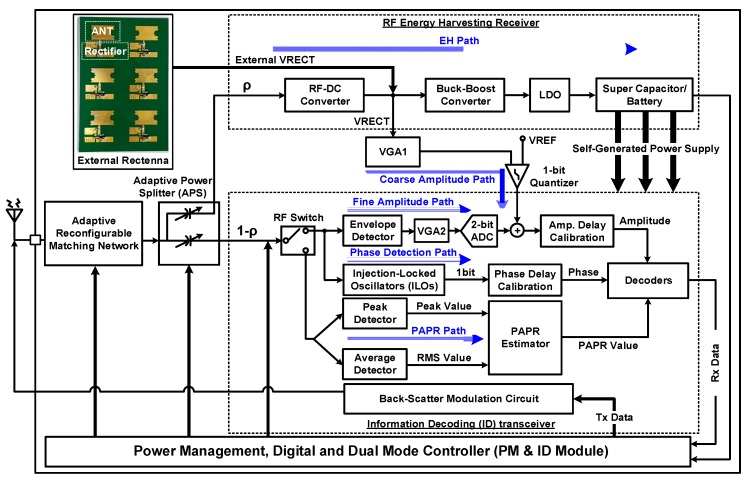
Top block diagram of the dual-mode SWIPT.

A receiver architecture comprises three paths—EH, ID, and PAPR paths—and a power management (PM)–ID control module, as shown in Figure 2. To harvest energy and decode information from the same signal at the receiver with self-powering, the duty cycle operation and the APS are adopted. The APS in front of the three paths splits the received signal with *ρ*, where 0 < *ρ* ≤ 1. The EH path is first activated to charge the battery. When the harvested energy is sufficient for self-powering and energy-neutral operation, either the ID path or the PAPR path is used, depending on the selected single-tone or multitone communication mode. We assume that infinitesimally small *ρ* is enough to achieve the required signal-to-noise ratio (SNR) for ID because of the property of the integrated receiver. Then, most of the signal power can be harvested at the EH circuit. The received signal after power splitting at the EH path can be expressed as

(1) $$y_{R}\left(t\right)=\sqrt{1-\rho }\left|h\right|s\left(t\right)+n\left(t\right)$$

where *s*(*t*) is the transmitted signal, *h* is the complex-valued channel gain with magnitude *|h|*, and *n*(*t*) is the channel noise modeled as additive white Gaussian noise (AWGN) with variance $\sigma^{2}.$ The received signal passes through the EH path during the EH block, which is described as $y_{EH}\left(t\right)=y_{R}\left(t\right)$, $t\in \left\{0,\frac{D_{n}}{D_{n}+1}T_{n}\right\},$ where *D_n_* is the duty ratio of EH to ID, and *T_n_* is the frame time during one duty cycle operation at the *n*th channel block. The harvested power $P_{EH}$ is evaluated as

(2) $$P_{EH}=\frac{D_{n}}{D_{n}+1}T_{n}\times \Psi_{EH}\left(P_{R}\right)\times \left(P_{R}\right)$$

where $P_{R}=E\left\{{\left|y_{EH}\left(t\right)\right|}^{2}\right\}=\left(1-\rho \right){\left|h\right|}^{2}.P_{R}$ (energy harvested from the noise power is ignored) is used for charging the battery and decoding information at the ID path as well. The received signal for ID is of the form $y_{ID}\left(t\right)=\sqrt{\rho }\left|h\right|s\left(t\right)+n\left(t\right)=y_{R}\left(t\right)$, $t\in \left\{\frac{D_{n}}{D_{n}+1}T_{n},T_{n}\right\}.$ Note that the time duration of the ID block is different from that of the EH block. The ID path is used to decode a single-tone waveform of the received signal with distinct energy levels. As the single-tone waveform is very sensitive to channel fading, we assume that channel estimation is performed before decoding, which consumes more circuit power relative to that of PAPR-based SWIPT. The exact energy level can be extracted by two steps. First, coarse and fine amplitudes are estimated from the EH and ID paths, respectively. After that, the exact energy level is jointly decoded such that the coarse amplitude saves the information during the EH block, and the ID path reads the information during the ID block. The phase can be measured through a low-power circuit injection-locked oscillator (ILO)-based demodulator. The phase information can be decoded once the energy level of the signal constellation is acquired. Hence, energy, coarse/fine amplitude, and phase paths are jointly combined for the proposed single-tone SWIPT. 

The PAPR path is used for PAPR-based ID. For this, the PAPR estimator simply measures the PAPR of the received signal envelope. The received PAPR can be evaluated as

(3) $$\mathrm{PAPR}=\frac{max_{t\in \left[0,T_{m}\right]}{\left|y_{ID}\left(t\right)\right|}^{2}}{\frac{1}{T_{m}}\int_{T_{m}}{\left|y_{ID}\left(t\right)\right|}^{2}dt}≅2N$$

Note that the symbol time of a multitone waveform is $T_{m}=T_{n}/(D_{n}+1)$. It consumes less power compared to the ID path because PAPR-based modulation does not require power-hungry devices, such as a mixer, voltage-controlled oscillator (VCO), and analog-to-digital converter (ADC), as well as channel estimation. Furthermore, the PCE in the low-power region is enhanced thanks to multitone waveforms. Thus, the PAPR path is suitable for a low-power consumption circuit while increasing the operational range with a lower rate. The PM–ID control module monitors the received power and controls the communication mode according to the PM–ID algorithm. For example, when the receive power is greater than the PM–ID threshold, the module feeds back the control message to the transmitter, and it activates the ID path for single-tone mode; otherwise, it activates the PAPR path for multitone mode [18,19,20].

### 2.2. Architecture

**Figure 3:**
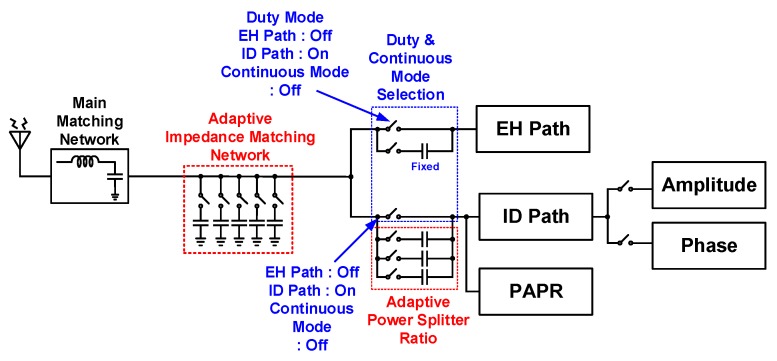
Adaptive impedance matching network and power-splitter blocks being controlled by field-programmable gate array (FPGA).

Figure 2 shows a block diagram of the proposed SWIPT. The proposed architecture is mainly divided into two paths: EH and ID paths. An antenna receives information carrying RF signals from the ambient environment and inputs to a matching network. As the signal strength of incoming RF signals varies, an adaptive impedance matching network was designed using the switches being controlled by field-programmable gate array (FPGA) to ensure maximum power transfer from the antenna to the power splitter, as shown in Figure 3.

The proposed APS distributes the incoming RF power between the EH and ID paths based on the input power level. As the input RF signal is not a constant quantity and its power level varies with time, the APS splits the received power between the EH and ID paths depending on the input power level. The proposed adaptive APS is shown in Figure 3. The APS deploys a large value capacitor on the EH path to transfer more power to the PM components, i.e., RF–DC converter, buck–boost converter, and low-dropout (LDO) regulator. In Figure 2, the distribution of RF input power is *ρ* to the EH path and (1 − *ρ*) to the ID path [9,10,19,20]. Dual-mode operation enables the proposed SWIPT system to work over a wide input power range while harvesting energy with higher PCE and conveys information simultaneously. Recent works on optimum signal design has revealed that using multitone waveforms [21,22] can bring considerable gain in the PCE compared to single-tone waveforms. These improvements result from building on a nonlinear model of the rectifier for energy harvesting, as clearly shown later in Figure 12a. In particular, multitone waveforms with higher PAPR increase the DC output of the rectifier [22]. However, the problem is that a simple modulation used for single-tone cannot be used on multitone waveforms. To circumvent this difficulty, a PAPR technique was proposed in the SWIPT system [15] to yield higher PCE and low-power ID. The PM–ID module, as shown in Figure 2, is implemented to monitor the ID and PAPR paths when single-tone and multitone waveforms are used. In the proposed architecture for dual-mode (single-tone and multitone) SWIPT, multitone transmission with high PAPR yields higher PCE than single-tone transmission when the received input power is low. In contrast, the latter yields higher PCE than the former when the received input power is high. The cross-over behavior of single-tone and multitone transmissions at different RF input power levels is discussed later. The cross-over point of PCE is shown to be −3 dBm.

The APS, shown in Figure 2 and Figure 3, splits the incoming power between the EH and ID paths. The EH path harvests the maximum energy and stores it in a supercapacitor that is later used to supply the ID path during duty cycle operation. The dual-mode controller turns on the RF switch for the amplitude path and phase path. Amplitude and phase detection in the ID path demodulates the information from the single-tone waveform and inputs to the information combiner block. 

The PAPR path remains off during single-tone operation. When the input power is low, multitone waveforms are used to boost up the PCE. During multitone operation, the EH path harvests energy from them and inputs to the coarse amplitude path. The harvested signal with information in it eventually reaches the adaptive dual-mode controller. As the signal strength of this waveform is limited, it is not sufficient to turn on devices of the amplitude and phase paths. Thus, the adaptive dual-mode controller turns on the single-pole, double throw (SPDT) switch for only the PAPR path. The PAPR path demodulates the multitone waveforms and inputs to adaptive dual-mode controller. The backscattering block inputs the measured data information to the antenna for uplink transmission.

To check the feasibility and the proof of concept, a board-level circuit for the proposed SWIPT system architecture was designed. This printed circuit board (PCB) uses commercial devices. OrCAD was used to design the schematic and PCB for the proposed architecture. It is a dual-mode SWIPT board operation operating at 900 MHz. Moreover, an off-chip (external) 5.2 GHz rectenna, shown in Figure 2, is connected to a buck–boost converter by a power metal–oxide–semiconductor (MOS) switch to check the operation of the RF EH receiver [23]. 

The RF energy harvester is implemented on the RF35-type board using commercial devices in order to maximize the power efficiency. A 6-stage rectenna is used, and its configuration is automatically selected depending on the input power level. The input range of the RF energy harvester is from 0 to +30 dBm, and the measured peak efficiency is 67% when the RF input power level at the RF–DC converter input node with respect to the air losses is +20 dBm at the frequency of 5.2 GHz. It can charge the IoT devices at a distance of 5.5 m with 48-array antenna charging station of transmit power 3.6 W [24].

### 2.3. Energy Harvesting Path

**Figure 4:**
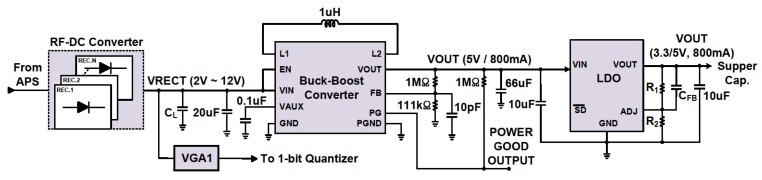
Block diagram of the proposed energy harvesting (EH) path.

Figure 4 shows a block diagram of the EH path. The EH receiver comprises an RF–DC converter, a buck–boost converter, and LDO blocks. The RF–DC converter is the first block at the input of the RF EH receiver to rectify the RF signal. A reconfigurable energy harvester is used, where multiple RF–DC converter circuits are implemented in parallel at high-input power level and in series at low-input power level. This brings higher PCE than the energy harvester with fixed multiple RF–DC converter circuits.

- Case I: High-input power level ($P_{R}>P_{R,opt.}$)

In this case, we have to divide the high-input power between the RF–DC converter circuits such that

(4) $$\frac{P_{R}}{N_{EH}}<P_{R,opt.}$$

where *N_EH_* is the number of RF–DC converter circuits depending on the received power. We need to design the RF–DC converter in a parallel structure.

- Case II: Low-input power level ($P_{R}\ll P_{R,opt.}$)

In this case, we have to power up the low-input power or use low *V_th_* devices such that

(5) $$P_{R}<P_{th,ON.}$$

We need to design the RF–DC converter in a series structure. In this study, to avoid PCE degradation, the proposed structure was made reconfigurable so that it could be controlled by FPGA both in parallel and in series. For example, in parallel structure, to find the optimal value of *N_EH_*, based on the measurement performance of a single RF–DC converter circuit, $P_{R,opt.}$ should be first determined. Then, based on the two conditions below, the optimum *N_EH_* can be determined as follows:

(6) $$\{\begin{array}{c} \frac{P_{R}}{N_{EH}-1}>P_{R,opt.}\\ \frac{P_{R}}{N_{EH}}<P_{R,opt.} \end{array}$$

Therefore, the PCE can be written as follows:

(7) $$\begin{array}{l} \eta =\overset{N_{EH}}{\underset{i=1}{\sum }}C_{i}N_{EH}f_{i}\left(\frac{P_{R}}{N_{EH}}\right)\\ \{\begin{array}{c} f_{i}\left(\frac{P_{R}}{N_{EH}}\right)=f\left(\frac{P_{R}}{N_{EH}}\right)\forall i\\ C_{i}=C\left(coupling\;loss\;and\;constant\right) \end{array} \end{array}$$

where $C_{i}$ is the coupling loss factor proportional to the number of RF–DC converter circuits (*N_EH_*). It can change the slope of the PCE curve and $P_{sat}$ a little bit. As a result, the simplified optimization problem for maximum PCE over the two factors $P_{R}$ and $N_{EH}$ can be formulated as follows:

(8) $$\eta \left(PCE\right):\underset{N_{EH}}{max}\;CN_{EH}f\left(x\right)$$

Based on the peak PCE of a single-stage RF–DC converter and the above equation, we can compute the maximum *PCE = P_sat_ = f*(*x*) for *x =* $\frac{P_{R,opt.}}{N_{EH}}$.

(9) $$\eta \left(PCE\right):\underset{N_{EH}}{max}CN_{EH}f\left(\frac{P_{R,opt.}}{N_{EH}}\right);0<C\leq 1\;and\;C\propto N_{EH}$$

To obtain the optimal PCE, we first determine *f*(*x*), which is proportional to $P_{R,opt.}$ and *N_EH_*. *N_EH_* can be determined by Equation (6). Also, to find the coupling loss factor value, which is proportional to *N_EH_*, the peak PCE of a single-stage RF–DC converter in a measured point is needed. Similarly, the series structure can be described with the necessary changes. A capacitor at the output of the RF–DC converter is also used as the low-pass filter to remove ripples at the output. Fast-switching transistors or diodes need to be used for the input signal of 900 MHz. The output of the RF–DC converter is fed to a buck–boost converter and variable gain amplifier (VGA1). In order to eliminate voltage variation, we used a buck–boost converter to get constant output voltage. When the input voltage level reduces due to the weak RF signals, the boosting operation of the buck–boost converter is performed to maintain the output voltage level and vice-versa. LDO is used to keep constant output voltage. A supercapacitor is connected at the output of the LDO to store energy and provides power for the ID transceiver and the charger of the battery.

**Figure 5:**
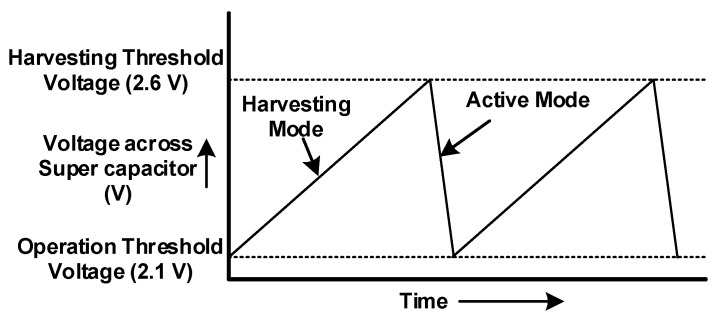
Voltage across supercapacitor and switching between modes.

Self-powering and energy-neutral operation in the SWIPT is very essential to provide power to the ID transceiver through the EH receiver, but this task is challenging. Energy harvested through the EH path is small compared to energy required for the continuous operation of the device, especially in batteryless applications. To overcome this situation, two device operational modes were designed: harvesting mode and active mode. During the harvesting mode, sufficient amount of energy is stored in the supercapacitor. All devices in the ID path are inactive during this mode. When harvested energy crosses the harvesting threshold voltage, the SWIPT system switches it to active mode. In the active mode, the SWIPT system will continue with its normal communication operation. When the stored energy drops below the operation threshold voltage, the ID path is disabled, and the SWIPT system is switched to the harvesting mode. This device operation and the voltage across the supercapacitor are shown in Figure 5. 

Switching the frequency between these two modes depends on the amount of RF energy available in the ambient source and the amount of energy required for the operation of the device. Higher ambient RF energy ensures quick charging of the supercapacitor and hence provides harvesting energy during the active mode. This will reduce the time when voltage across the supercapacitor goes below the operation threshold voltage, and the SWIPT system switches to the harvesting mode. Thus, it reduces the overall device switching frequency between the two modes.

The amount of energy required for the operation of the device is also very critical in reducing mode-switching frequency. An increase in the required energy will quickly discharge the supercapacitor, forcing the SWIPT system to switch to the harvesting mode. Devices that require low operational energy draw a very small amount of energy from the supercapacitor. If the required amount of energy for the device operation is equal to or less than the amount of energy being harvested, then the device can operate continuously without switching to the harvesting mode.

### 2.4. Information Decoding Path and PAPR Path

**Figure 6:**
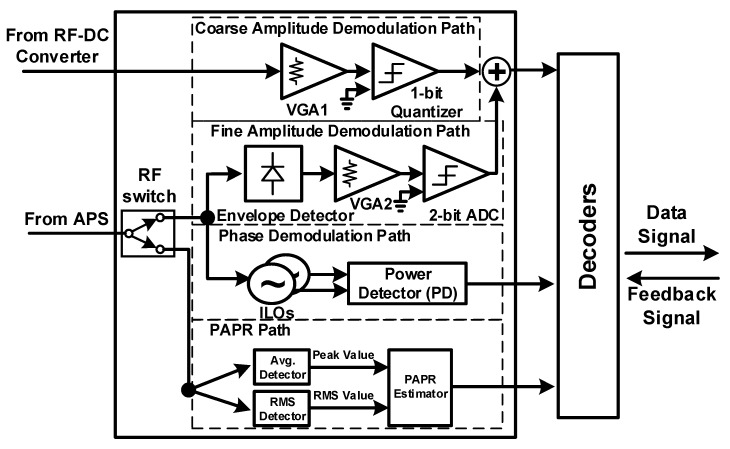
Block diagram of the proposed information decoding (ID) path.

The ID path consists of four main paths—coarse amplitude detection, fine amplitude detection, phase detection, and PAPR path—as shown in Figure 6. The coarse amplitude detection path includes the RF–DC converter of the EH path, a VGA1 with a 1-bit quantizer. With the fine amplitude detection path, the signal is passed through the envelope detector (ED). It consists of a diode used for rectification and a RC low-pass filter to recover the message signal. The message signal gain is then adjusted through VGA2. A 2-bit ADC is used to convert the message signal into binary form. The output of the 2-bit ADC in the fine amplitude path is combined with the output of the 1-bit quantizer in the coarse amplitude path and delivered to the decoders after adjusting the delay between the paths.

**Figure 7:**
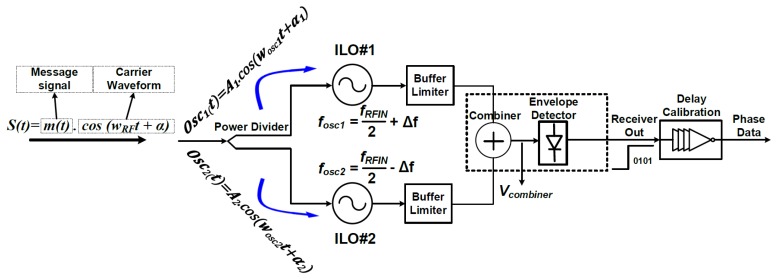
Operating principle of phase detection path.

The phase detection path includes two parallel demodulators to extract ASK and binary phase-shift keying (BPSK) message signals. A low-power ILO-based demodulator is used to demodulate BPSK message signals. It consists of a RF power divider, ILOs that have two inductor–capacitor (LC) oscillators (ILO#1 and ILO#2), and a power detector (PD) stage, including a combiner and the envelope detector. The power divider performs two main tasks. The first is to provide the same signal power to both oscillators, and the second is to provide isolation between the two oscillators. For correct demodulation, ILOs must be tuned to nearly half of the input RF signal, and Equation (10) must be satisfied.

(10) $$f_{OSC1}<\frac{f_{RFIN}}{2}<f_{OSC2}$$

where *f*_OSC1_ and *f*_OSC2_ are free-running frequencies of the two oscillators in the ILOs. *f_RFIN_* is the frequency of the RF input signal. Figure 7 shows the operating principle of the phase detection path. The phase changes in the input signal, *S*(*t*), are detected through ILOs. In the following equation, the power divider at the *V_combiner_* node shows that the input signal has 0° phase shift:

(11) $$V_{combiner}=2A.cos\frac{\alpha_{1}}{2}.cos\frac{\omega_{RF}}{2}t$$

When the phase of the input signal changes in “π”, the output phase of ILO#1 changes in $+\frac{\pi }{2}$ (due to higher free-running frequency), while the output phase of ILO#2 changes in $-\frac{\pi }{2}$ (due to lower free-running frequency). Thus, the output of the combiner is given as follows:

(12) $$V_{combiner}=2A.sin\frac{\alpha_{1}}{2}.cos\frac{\omega_{RF}}{2}t$$

By comparing Equations (11) and (12), the phase change in the injected BPSK signal is manifested at the combiner output as the amplitude changes from $2A.\cos\frac{\alpha_{1}}{2}$ to $2A.\sin\frac{\alpha_{1}}{2}$. It can be demonstrated using the above discussion that the later phase shift of the BPSK signal leads to the amplitude flipping between these two statuses. Therefore, the BPSK signal is converted to the ASK signal by these two ILOs. After that, an ED is added to demodulate the ASK signal to baseband so that BPSK demodulation is accomplished.

**Figure 8:**
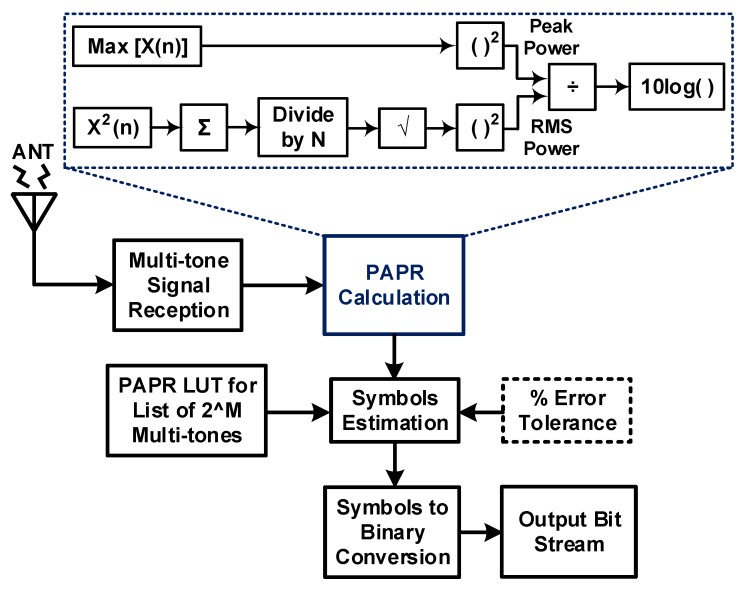
Block diagram of the proposed peak-to-average power ratio (PAPR) path.

A PAPR-based modulator/demodulator provides a simple and efficient way to transmit/receive information [18,19,20]. A block diagram of a PAPR demodulator is shown in Figure 8. The PAPR value of a received multitone signal is calculated by the PAPR calculation block, as explained in detail in the system model description. The PAPR value of a symbol can be calculated as follows: $PAPR_{symbol}=\frac{Peak\;Power_{symbol}}{Average\;Power_{symbol}}.$ The transmitter (modulator) details are omitted due to space limitation.

### 2.5. Backscatter Modulator

Backscatter modulation is used to transmit the message signal back to the transmitter when power is limited to power up the ID transceiver. The PM–ID module will control the switch, which shunts the antenna output to perform modulation to transmit message data. Using a modulator with backscatter technique, the signal is transmitted without any oscillators. Therefore, the power consumption is considerably less and equal to 260 nW.

## 3. Simulation and Experimental Results

**Figure 9:**
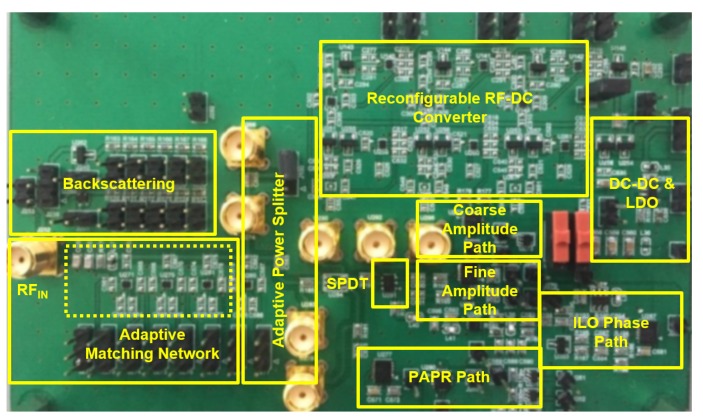
Measurement board of 900 MHz dual-mode SWIPT system.

To validate our proposed SWIPT architecture, SWIPT PCB (31 mil Rogers RO4003C substrate) was designed, as shown in Figure 9, with multitone SWIPT board at 900 MHz. 

**Figure 10:**
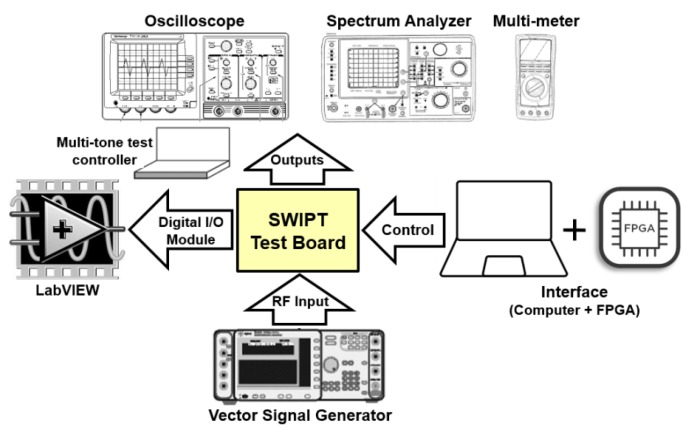
Measurement environment of 900 MHz SWIPT system.

**Figure 11:**
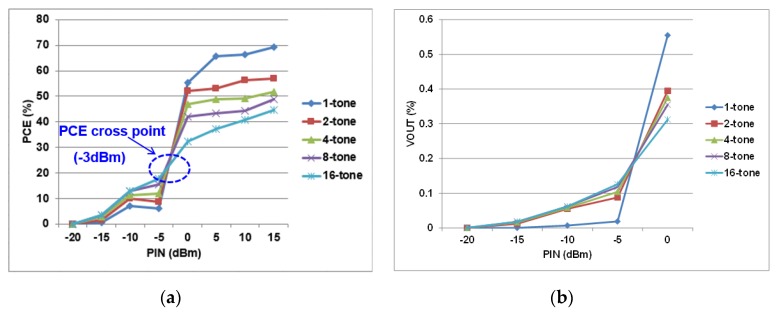
Measured performance of a single-stage radio frequency–direct current (RF–DC) converter. (**a**) Power conversion efficiency (PCE) versus input power *P*_IN_ with respect to number of tones; (**b**) output voltage versus input power *P*_IN_ with respect to number of tones.

Figure 10 shows the experimental setup. Adaptive matching network was used in the PCB to match the point. The S11 (reflection coefficient or return loss) of −15.73 dB was measured at 900 MHz. Figure 11 shows the measured results of a single-stage RF–DC converter of the EH path. In Figure 11a, the PCE was measured against the RF input power with respect to the number of tones. For low-input power level, multitone had a better PCE performance compared to single-tone. As the input power level increased, single-tone became more dominant with better performance than multitone. At −10 dBm input power level, 16-tones had the maximum PCE of 12.8% for the single-stage RF–DC converter. On the other hand, single-tone had the maximum power PCE of 69% at 15 dBm input power level. All tones tended to have the same PCE at −3 dBm input power level, which is called the PCE cross point. Figure 11b shows the output voltage V_OUT_ versus input power level with respect to a number of tones.

**Figure 12:**
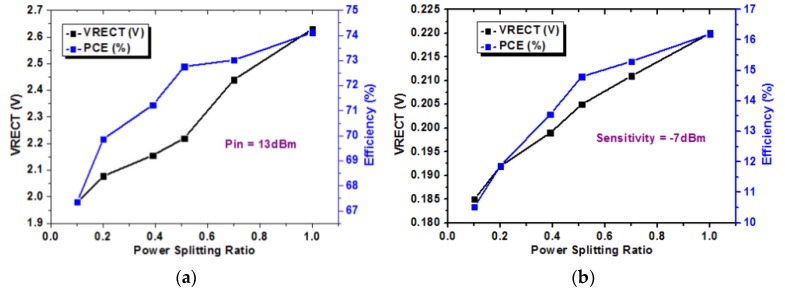

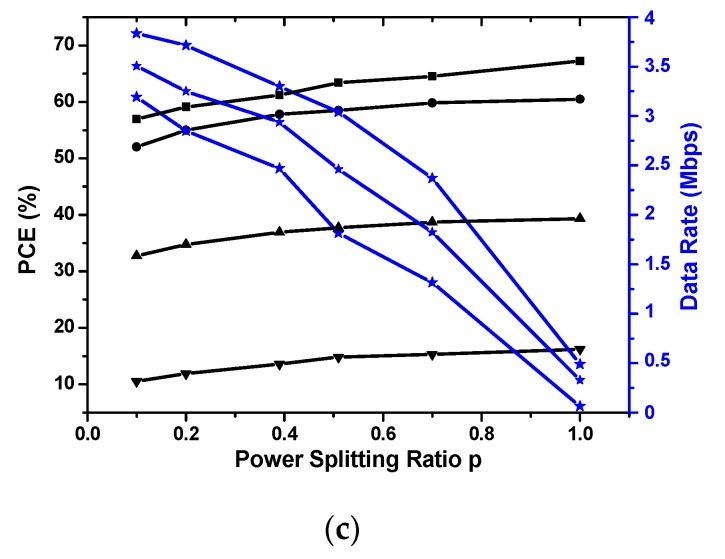
Measured PCE and output voltage of the rectifier (*V*_RECT_) of a single-stage RF–DC converter with respect to the power-splitting ratio when the input power level was (**a**) +13 dBm and (**b**) −7 dBm. (**c**) The measured PCE and data rate with respect to the power-splitting ratio at different input power levels.

Figure 12a–c shows the measured results of a single-stage RF–DC converter for different PS ratios (*C_EH_*/*C_ID_*) between the EH path and the ID path. *C_EH_* and *C_ID_* are the capacitors used on the EH path and the ID path, respectively. Figure 12a,b shows the PCE and the output voltage of the rectifier (*V*_RECT_) versus the power-splitting ratio at 13 and −7 dBm input power level, respectively. By increasing the PS ratio, PCE and V_RECT_ increased constantly. Figure 12c shows the PCE and data rate versus the PS ratio at different input power levels. After converting the input RF signal into DC through a RF–DC converter, a buck–boost converter was used to provide a stable output voltage. The measured output voltage of the buck–boost converter was 5.19 V. Also, LDO was used to maintain an output voltage of 3.29 V. For ID, a multilevel BPSK RF signal was generated through a vector signal generator. To extract the amplitude-modulated information, a signal was applied at the input of the envelope detector at the amplitude path. The gain of input signal was adjusted to make it suitable for applying at the input of 2-bit ADC. 

**Figure 13:**
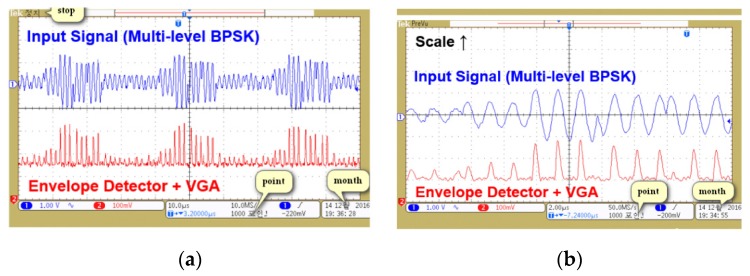
Measured (**a**) full-scale and (**b**) magnified waveforms of envelope detector and variable gain amplifier (VGA2).

Figure 13a,b shows the output of the ED after the RF–DC converter operation. Signal gain was controlled through VGA2. The output of ED was applied at the input of 2-bit ADC, which consisted of three comparators with reference voltages of 157.5, 148.5, and 145.2 mV, respectively. Three comparator outputs were encoded into a 2-bit ADC code by applying a logical operation in LabVIEW. Comparator output was read through NI 6555 high-speed digital input/output (I/O) module. Encoding was performed through one XOR and two AND gates to generate a 2-bit ADC code.

**Figure 14:**
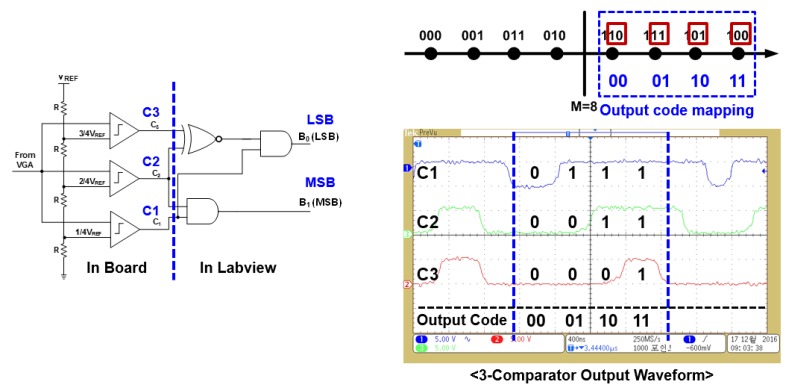
Measured digital encoding waveforms using LabVIEW (The meaning of the Korean is the same as they are in Figure 13).

Figure 14 shows a 2-bit encoding process. These encoded 2-bit symbols were verified through a constellation used for modulating digital data. Each symbol consisted of three bits, with two bits representing the amplitude variation, and the other one indicating 180° phase change in a modulated signal. The 3-bit symbol was recovered by combining the output of a 2-bit ADC along with phase variation information through ILOs.

**Figure 15:**
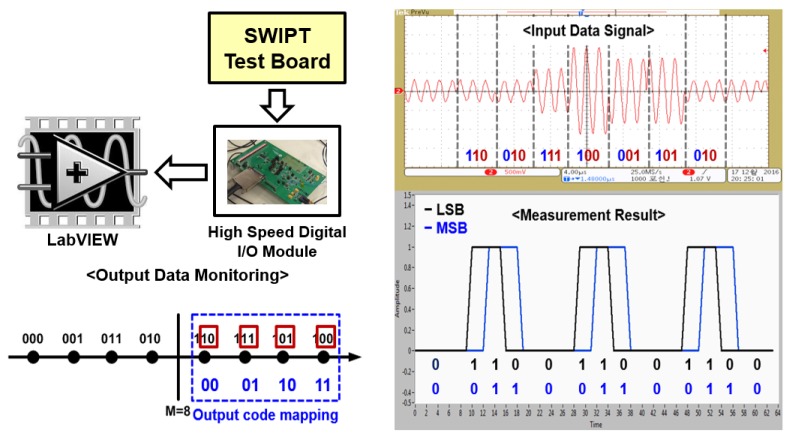
Measurement results of 900 MHz SWIPT amplitude detection path (The meaning of the Korean is the same as they are in Figure 13).

**Figure 16:**
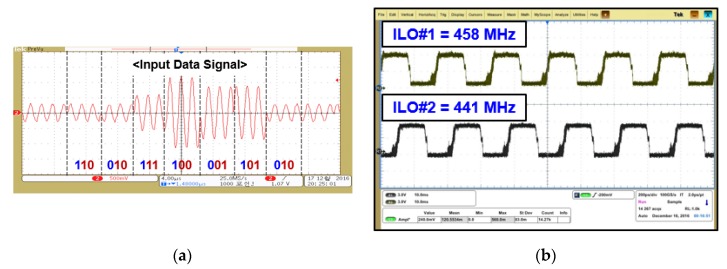
(**a**) Input data signal of phase detection path and (**b**) free-running frequencies of injection-locked oscillator (ILO) (The meaning of the Korean is the same as they are in Figure 13).

Figure 15 shows a modulated signal through a constellation symbol map that recovered 2-bit amplitude information according to the transmitted symbols. Phase change in the input signal was detected through ILOs. It consisted of two oscillators—ILO#1 and ILO#2—free-running at 458 MHz and 441 MHz, respectively. Phase changes in the input signal as per constellation are shown in Figure 16a,b, respectively. ILOs output single-bit information for about 180° with a change in the input signal.

**Figure 17:**
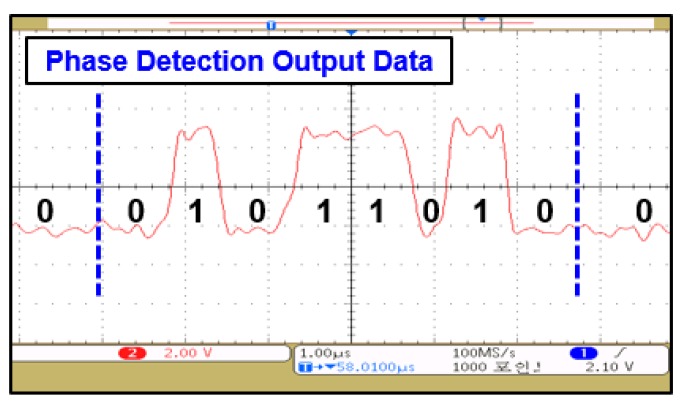
Measured output data of phase detection path (The meaning of the Korean is the same as they are in Figure 13).

This change in the output of ILOs is shown in Figure 17. By combining this ILO output along with the 2-bit ADC output, we successfully demodulated transmitted information. For measuring PAPR value, multitone signal was generated through a vector signal generator. The peak and average power values of the input signal were calculated through the peak and average power detection circuit.

**Figure 18:**
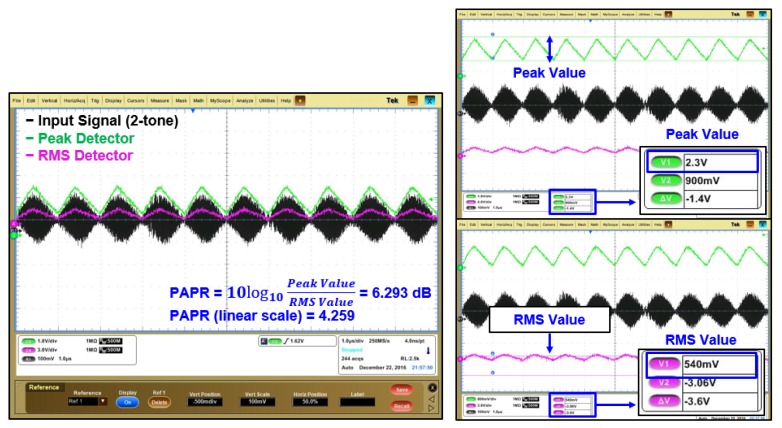
Measurement results of 2-tone PAPR path.

Figure 18 shows the peak and average power values of 2.3 V and 540 mV, respectively, for a 2-tone input signal. The PAPR value was calculated through linear and logarithmic formula, giving PAPR values of 4.259 and 6.293 dB, respectively. To date, there has mostly been theoretical research on SWIPT. Table 1 summarizes the performance of the proposed state-of-the-art SWIPT with hardware implementation and experiments. To the best of our knowledge, this is the first time it has been done. 

## 4. Conclusions

This paper proposes a self-powered SWIPT architecture relying on RF energy harvesting in low-power IoT devices. The proposed architecture enables self-powering for energy-neutral operation and adopts the power-splitting scheme to ensure duty cycle-based, dual-mode operation with adaptive PM–ID operation. This will lead to sustaining a battery-free IoT sensor network for self-powered devices. 

The architecture employs a high-efficiency RF–DC converter to convert RF energy into DC voltage. A buck–boost converter and LDO both secure further stable output voltages for self-powering. The information decoding path comprises amplitude, phase, and PAPR estimation. The envelope detector along with a 2-bit ADC decodes amplitude-modulated information, while an ILO-based BPSK demodulator is used to extract phase-encoded information. To validate the SWIPT architecture, the PAPR value of incoming signal was measured through its peak and average power values. The results showed high performance of −7 dBm sensitivity with 69% efficiency. 

This design provides a promising solution for future IoT devices while extending the operational range via the dual-mode operation.

## Figures and Tables

**Table 1:** Performance summary of the SWIPT system.

Parameters	Value
**Technology**	PCB level
**Supply (V)**	3.3/5
**Maximum EH efficiency (%)**	69 for 300 Ω load
**Frequency band (MHz)**	900 (/5.2 GHz using rectenna)
**Sensitivity (dBm)**	−7 dBm
**Receiver modulation**	ASK/BPSK/PAPR
**Data rate (Mbps)**	1/2.5/4
**PAPR support**	Yes
**Implementation**	PCB
**SWIPT support**	Yes
**Power-splitting ratio Transmitter modulation**	0.44 backscatter
